# Computational Fluid Dynamics (CFD)-Based Optimization of Injection Process during Endoscopic Mucosal Therapy

**DOI:** 10.3390/bioengineering7040136

**Published:** 2020-10-27

**Authors:** Mohamad Aghaie Meybodi, Rohit Saini, Amirfarhang Mehdizadeh, Reza Hejazi

**Affiliations:** 1 Division of Gastroenterology, Department of Internal Medicine, Kansas University Medical Center, Kansas City, KS 66160, USA; m.aghaie.meybodi@gmail.com; 2 School of Computing and Engineering, Civil and Mechanical Engineering Department, University of Missouri Kansas City, Kansas City, MO 64110, USA; rohitsaini@mail.umkc.edu

**Keywords:** computational fluid dynamics, endoscopic mucosal therapy, lifting characteristics, injection dynamics

## Abstract

Creation of a submucosal plane to separate the lesion from the deeper muscle layer in gastrointestinal tract is an integral and essential part of endoscopic resection therapies such as endoscopic mucosal resection (EMR) and endoscopic submucosal dissection (ESD). Thereby, an optimized submucosal injection technique is required to ensure a successful process. In this study, the computational fluid dynamics (CFD) technique is employed as a foundational step towards the development of a framework that can provide useful directions to optimize the injection process. Three different lifting agents, including Glycerol, Eleview^®^, and ORISE^®^ gel have been used for this study. The role of different injection angles, injection dynamics, and effect of temperature are studied to understand the lifting characteristic of each agent. The study shows that Eleview^®^ provides the highest lifting effect, including the initial injection period. To evaluate the impact of the injection process, two cases are simulated, termed static injection and dynamic injection. Under static injection, the injection angle is investigated from lower to higher angles of injection. In the dynamic injection, two cases are modulated, where a continuous change of injection angle from lower to higher degrees (denoted as clockwise) and vice-versa in the anti-clockwise direction are investigated. Increased lifting characteristics are observed at decreasing/lower angle of injection. Further, the correlation between temperature of the lifting agents and their lifting characteristics is investigated.

## 1. Introduction

Colorectal cancer has been the third most diagnosed cancer worldwide in recent years. Colorectal and stomach cancer were the second (9.2%) and third (8.2%) leading causes of cancer death in 2018, respectively [1]. Early endoscopic detection and treatment of precancerous lesions and early-stage cancer could lead to a decrease in cancer incidence and mortality rate [2,3].

Endoscopic mucosal resection (EMR) and endoscopic submucosal dissection (ESD) are advanced endoscopic techniques developed for the management of sessile and flat polyps and neoplasms confined to the mucosa or the superficial submucosa of the gastrointestinal (GI) tract [4,5,6]. The major adverse events of these minimally invasive procedures are bleeding, perforation, and inadequate polypectomy [7,8].

**Figure 1:**
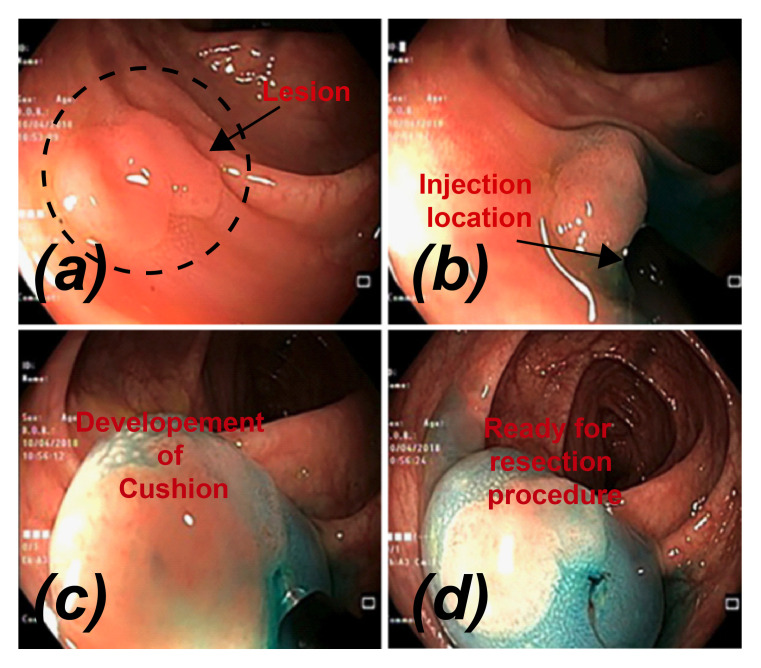
Procedure of submucosal injection of a colonic flat lesion [10].

Figure 1 chronologically describes the procedure of submucosal injection in case of flat lesion before EMR or ESD. The procedure commences with injecting a solution between the lesion and the muscle layer, resulting in the development of the safety cushion [9]. Methylene blue, inert dye resulting in blue color, is added to lifting agents (like ORISE^®^ and Eleview^®^) for better visibility of lesion and submucosal surface [10]. This cushion facilitates the complete and or en bloc resection and is also expected to decrease the procedure mechanical and thermal adverse events [5,11]. Several solutions have been used for creating a submucosal cushion. Normal saline is extensively available and often injected, but it is rapidly absorbed, which increases the need for re-injection and prolongs the procedure [12]. More viscous solutions were shown to be more effective and, therefore, resulting in a higher rate of complete resection resulting in a lower rate of residual tissue and recurrence of pre-cancer lesion [13]. However, there are still many uncertainties about the optimal injection solution and procedure that can provide the best lifting properties, duration of lifting and ease of use.

The overarching goal of the present study is to employ the computational fluid dynamics (CFD) technique as a reliable tool towards the development of a framework that can provide useful directions to optimize the injection process. Currently, CFD methods are used for a wide range of biomedical engineering and medicine, particularly in cardiovascular engineering. They are used to enhance predictive capabilities and moving toward patient-specific precision medicine [14]. It further proved to be a reliable predictive tool for pharmaceutical aerosol deposition in lung and nasal cavity [15,16].

## 2. Materials and Methods

CFD is the science that allows for solving equations of fluid motion to produce quantitative and/or qualitative predictions of fluid flow phenomena that otherwise are not possible. There are some considerations and steps that need to be followed to formulate a reliable CFD simulation. These include designing an appropriate geometry that best resembles the major and essential properties of the real geometry. The next step is to discretize the geometry into several small elements that can be created by various mesh generation methods. CFD uses numerical techniques to solve the conservation laws (conservation of mass and momentum) for each of those small elements. There are several techniques for mesh generation, some of which are fully automatic, i.e., once the geometry is provided, the mesh will be generated typically with a pre-defined resolution. Mesh can be refined (increase in number of elements) in order to achieve higher accuracy, if necessary. However, automatic mesh generation is usually successful in the case of simple geometries using structured mesh generation algorithms. Dealing with complex geometries usually requires a combination of structured and unstructured meshes that generally need to be done manually. The next step is to define appropriate boundary conditions such as inlet, solid surface, and outlet based on the situation. CFD solver then produces the pressure and velocity field over all elements at each time step.

More importantly, federal regulatory agencies such as the US Food and Drug Administration (FDA) have recognized the capability/potential of CFD and are involved in formulating verification and validation (V&V) guidelines to help fast-track the FDA approval process of medical devices that use CFD during their research and development (R&D) phase [14].

The primary aim of this study is to investigate the capability of CFD to capture the general dynamics of the injection process by designing a simple model/geometry that can be used to study the fluid movement dynamics such as injection angle, material properties, and temperature. The secondary goal is to create a foundational basis/framework that can be extended to predict more complex phenomena moving towards the real situation.

## 3. Computational Modeling

### 3.1. Governing Equations

CFD package of ANSYS, Fluent Software [17] has been used to solve the Navier-Stokes equations to simulate the transient dynamics of the submucosal injection process. The governing equations for the incompressible two-phase flows are solved in the conservative form:

(1) ▿·U=0

(2) $$\frac{\partial \rho \vec{u}}{\partial t}+▿\cdot (\rho \vec{u}\vec{u})=-▿P+▿\cdot \left[\mu (▿\vec{u}+{\left(▿\vec{u}\right)}^{T})\right],$$

where ρ, *P*, μ and *u* are fluid density, pressure, dynamic viscosity, and velocity, respectively. The fluid is modeled as a single mixture. Further, the Volume-of-Fluid (VOF) interface capturing method was used for tracking and locating an interface location to determine the lift achieved by each agent during the injection process. Under the VOF method, the primary phase (still phase) is defined in computational cells (elements) as volume of fraction (alpha) = 0, and the secondary phase (injected fluid) is defined in computational cells as alpha = 1. The cells where (alpha) is between 0 and 1 are those that are split by the interface (include both phases) and identified by solving the following equation:

(3) $$\frac{\partial \alpha }{\partial t}+▿\cdot \left(\alpha \vec{u}\right)=0.$$

Furthermore, it was assumed that the flow remains laminar throughout the entire process. It should be mentioned here that the primary goal of the simulations is to investigate (1) the capability of CFD techniques in dealing with the complex multi-phase phenomena during the injection process and (2) to gain initial insights into the dynamics of the process. Therefore, the process has been simplified to a two-dimensional computational domain with impermeable walls geometry.

### 3.2. Lifting Agents

Three different lifting agents (Glycerol, Eleview^®^, and ORISE^®^ gel) have been used for this study. Glycerol is a hypertonic solution containing 10% glycerin and 5% fructose dissolved in a Normal Saline [18] (Glycerol does not contain methylene blue). Eleview^®^ (Aries Pharmaceutical, San Diego, CA, USA) is the FDA approved submucosal injection solution. It was composed of water, sodium chloride, poloxamer 188 (bulking and structural agent), polyoxyl-15-hydroxystearate (emulsifier), and medium-chain-triglycerides (oil component). Methylene blue was added to improve visibility. As a ready-to-use, sterile, premixed composition, it is a convenient option for clinicians. ORISE^®^ (Boston Scientific, Marlborough, Massachusetts) is another ready-to-use solution that received FDA approval to be used in the entire gastrointestinal tract, recently [10]. It is worth noting that ingredients of ORISE gel is similar to Eleview^®^ gel [10]. However, it seems that fraction of each ingredient for ORSIE^®^ gel is different as compare to Eleview^®^, which leads to different fluid/material properties such as density, visocisty, etc., relevant for the present investigation.

### 3.3. Flow Geometry and Boundary Conditions

**Figure 2:**
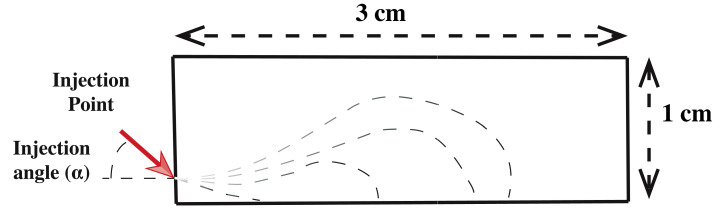
Schematic of the two-dimensional computational domain. Dash lines replicates the development of cushion.

Impermeable wall boundary condition was applied on all the boundary walls. The diameter of the injection point that mimics the lumen is 0.6 mm. A velocity profile is imposed at this location using the Velocity Inlet Boundary Condition to simulate the liquid leaving the lumen in a real situation. The two-dimensional schematic of the computational domain is shown in Figure 2.

## 4. Results

For all simulations, the total injected mean volume was 1 mL for 0.2 s. In this modeling, we studied the role of different injection angles, injection dynamics, and the effect of temperature on each agent’s lifting characteristic.

Three agents were compared by simulating the injection with 30 degrees. During the initial injection period (0.1 s), Eleview^®^ provides the most lifting effects (8.18 mm). ORISE^®^ and Glycerol provided 7.61 mm and 7.24 mm lifting, respectively. After continuing injection for 2 s, Eleview^®^ remained the most effective agent (9.57 mm). However, ORISE^®^ and Glycerol reached the same height (9.26 mm), as shown in Table 1.

### 4.1. Static Injection

**Figure 3:**
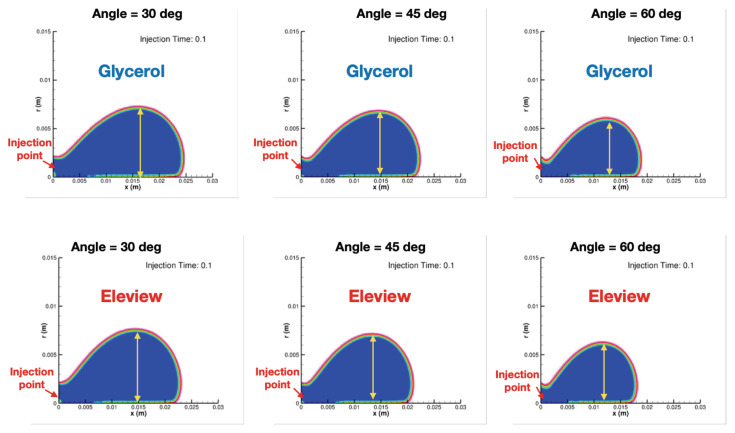
Comparison of two gels in static injection.

To evaluate the static injection, the injection of Eleview^®^ was modulated in 30, 45, and 60 degrees. In the 0.1 s (injection initiation), the 8.1 mm cushion developed in a 30-degree angle, whereas the lifting due to injection in 60 degrees was less than 6.5 mm, as shown in Figure 3. The final lifting height was 9 mm vs. 7.8 mm in 30 and 60 degrees, respectively. The evaluation of the static injection with Glycerol provided comparable results.

An ideal lifting agent should provide long-lasting maximum lifting duration as well as hemispherical shape to facilitate successful snaring [18]. Sumiyoshi et al. [19] observed a hemispherical shape of the cushion using Glycerol as a lifting agent and measured the maximum cushion lift of around 5 mm after injection. From Table 1, it can be seen that Glycerol at 60 degrees provides approximately similar maximum lift at the initial period of injection, along with hemispherical shape development using all three lifting agents (shown in Figure 3). Therefore, it confirms that CFD can capture the general dynamics of the injection process in a qualitative, as well as quantitative manner, even when using simple geometric configuration.

### 4.2. Dynamic Injection

**Figure 4:**
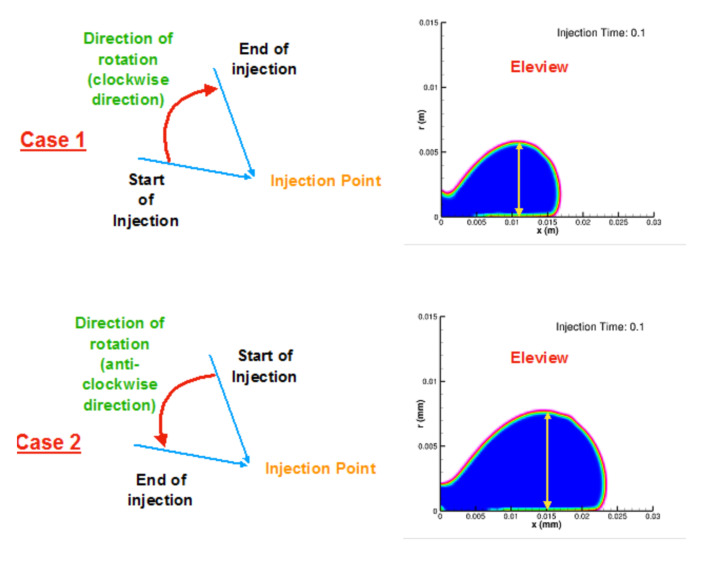
Comparison of polyp formation in dynamic injection.

Two cases were modulated to assess the dynamic injection. First, the angle of injection increased from 20 to 70 degrees (clockwise) in the interval of 0.05 s, till 0.3 s of flow time. Second, the angle decreased from 70 to 20 degrees (counter-clockwise) in the same flow time. In the first case, the lift height decreased with an increasing angle of injection, while the lifting height increased in the second case and can be seen in Figure 4. Dynamic injection with case 2 provides an approximately equal amount of lift as compared to static injection in 30 degrees angle.

### 4.3. Temperature

**Figure 5:**
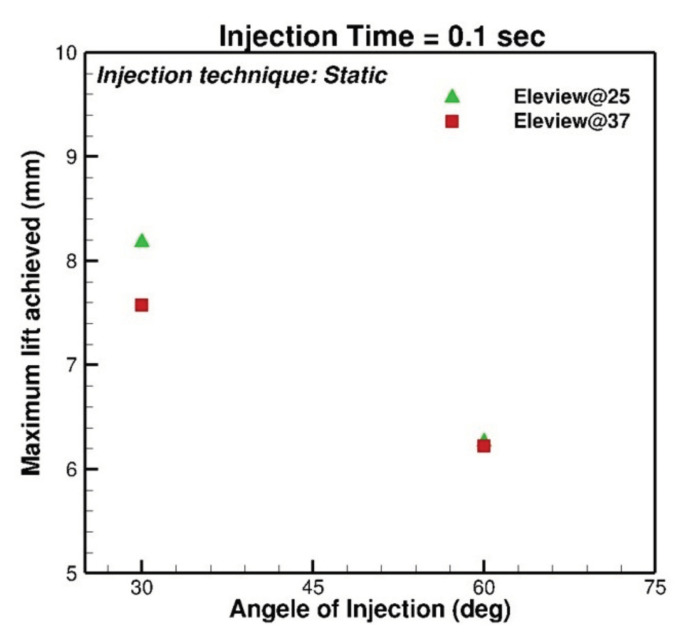
Comparison of Eleview^®^ gel at $25{\;}^{\circ }$C and $37{\;}^{\circ }$C.

Eleview^®^ injection also modulated in two different temperatures (25 °C and 37 °C) to evaluate the effect of temperature on the lifting agents and consequently on the maximum lift. It was expected that increasing temperature reduces the viscosity of the fluid, and hence, the maximum lift is reduced. The computer modulation showed it only happened at a lower injection angle (30 degrees) and it did not have a significant effect at a higher angle (60 degrees), which can also be seen in Figure 5.

## 5. Discussion

Minimal invasive endoscopic procedures such as EMR and ESD are approved as a treatment of dysplastic and early cancerous lesions limited to the mucosa and submucosa without lymph node involvement [20,21]. Mucosal elevation through the lifting agent helps decrease the complication rate and increases the chances of resection without remaining. The ideal injection solution should provide a sustained high submucosal cushion for safe and effective endoscopic resection and also preserving the tissue sample for pathologic assessment [22]. Hyun et al. [23] studied different lifting agents (Normal saline, Mannitol, Sodium Hyaluronate, Hydroxypropyl Methylcellulose, and Fibrinogen) on a fresh transverse colon specimen. They showed the initial heights after injection were comparable for all agents. However, agents with higher viscosity have a higher height after 10 min.

In this study, we simulated the submucosal injection to compare different solutions and optimize the injection procedure. The injection of 3 common lifting agents was investigated at different modes of injection (static and dynamic) and the effect of temperature. In our experiment, Eleview^®^ demonstrated the most effective lifting from the injection initiation to termination in all models. In the next step, the static injection was stimulated to assess the effect of the injection angle on the submucosal cushion. In static injection, the rate of development of lifting was faster at lower angles compared to higher angles, which led to the early formation of a cushion. Injection at a lower angle provided enough height cushion with less solution injection.

Injecting in the low angle (30 degrees or less) is technically troublesome and often impossible. Dynamic injection by changing the angle during injection was modulated to overcome this problem. Starting injection at a high angle (70 degrees) and decreasing along with injecting, provides the cushion height comparable to static injection at a low angle. The dynamic injection can lead to reduce the requirement of re-injections and consequently reduce the procedure time.

The temperature difference between ex-vivo and in-vivo (25 ${}^{\circ }$C vs. 37 ${}^{\circ }$C) is the biggest concern in the investigation of a new submucosal injection solution. Therefore, we stimulated the Eleview^®^ injection in both temperatures. It showed only static injection at a low angle can be affected by the lower temperature. This module can be used to develop new lifting agents in the future to have more accurate estimation before applying the new agents in animals or humans. To our knowledge, this is the first report of using computational fluid dynamics to simulate lifting in submucosal endoscopic therapy and could be a framework for further use of this methodology for simulating and drug delivery in other GI procedures such as cyst injection/drainage and radioisotope delivery. The geometry, although simple, can provide useful clues on the dynamics of the flow during the injection process. Understanding the dynamics of the process at this fundamental/simple level is necessary for further incorporation of different features such as permeability in order to best resemble the real situation. The main limitation of our study is modulation in the standard situation without considering the other properties of procedure such as permeability, motion effects, size and depth of polyp, etc. We are currently developing a more accurate modeling of submucosal fluid dynamics by incorporating more variables. The ultimate goal will be to apply our module to evaluate the lifting agents and optimize the procedure before animal and human experiments.

## Figures and Tables

**Table 1:** Variation of maximum lift achieved at 30 degrees.

	30 Degrees			60 Degrees		
	Eleview	Orise	Glycerol	Eleview	Orise	Glycerol
0.1 s	8.1814 mm	7.6182 mm	7.2428 mm	6.2480 mm	6.3075 mm	6.0108 mm
0.15 s	8.9531 mm	8.6402 mm	8.1605 mm	7.2356 mm	7.2502 mm	6.9108 mm
0.2 s	9.5788 mm	9.2659 mm	9.2659 mm	7.8217 mm	7.8535 mm	7.4495 mm
